# Rewriting the dendritic cell code in cancer—from subset identity to immunotherapeutic design

**DOI:** 10.1002/1873-3468.70108

**Published:** 2025-07-16

**Authors:** Estevão Carlos Silva Barcelos, Doriana Ricciuti, Giada Mondanelli, Marco Gargaro

**Affiliations:** ^1^ Department of Pharmaceutical Sciences University of Perugia Italy; ^2^ Department of Medicine and Surgery University of Perugia Italy

**Keywords:** cancer immunotherapy, dendritic cell subsets, single‐cell transcriptomics, spatial mapping, tumor microenvironment

## Abstract

Dendritic cells (DCs) are central orchestrators of antitumor immunity, bridging innate sensing with adaptive T‐cell responses. This review dissects the developmental pathways and functional specializations of diverse DC subsets—including cDC1, cDC2, pDCs, DC3s, tDCs, moDCs, and emerging RORγt^+^ antigen‐presenting cells (APCs)—and explores how the tumor microenvironment (TME) dynamically reprograms these cells. Immunosuppressive cytokines, metabolic stress, hypoxia, and altered lipid metabolism can induce tolerogenic phenotypes such as mregDCs and ISG^+^ DCs, dampening antigen presentation and T‐cell activation. We detail how specific DC subsets interact with the TME—either reinforcing immune evasion or promoting antitumor immunity—depending on their transcriptional states and spatial organization. Emphasis is placed on recent findings from spatial transcriptomics and single‐cell studies that reveal key DC–T cell niches critical for immune control. Furthermore, we evaluate current and emerging therapeutic strategies that aim to restore DC functionality or exploit their antigen‐presenting capabilities, including mRNA vaccines, receptor‐targeted delivery, CD40 agonists, and *in situ* cellular reprogramming. By integrating mechanistic insights with clinical advances, this review underscores the potential of context‐aware, subset‐specific DC interventions to overcome immune suppression and enhance cancer immunotherapy.

**Impact statement**

By dissecting the complexity and plasticity of dendritic cell subsets in cancer, our work offers novel insights into their reprogramming by the tumor microenvironment and therapeutic exploitation, paving the way for next‐generation, context‐aware immunotherapies.

## Graphical abstract

Dendritic cells (DCs) play central roles in cancer immunity but are often subverted by the tumor microenvironment. This review explores the diversity of DC subsets, their functional plasticity, and emerging therapeutic strategies to reprogram DCs for enhanced antitumor responses, including vaccines, *in vivo* targeting, and DC‐based immunotherapies. Created in BioRender. https://BioRender.com/smidtu6.

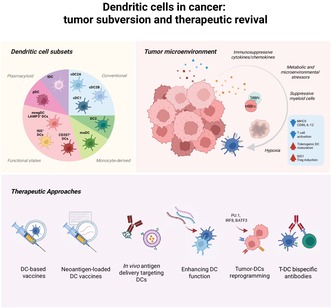

## Abbreviations

**ADORA1**, adenosine A1 receptor

**AhR**, aryl hydrocarbon receptor

**APC**, antigen‐presenting cell

**BiCE**, bispecific T cell engager

**BTLA**, B and T lymphocyte attenuator

**C/EBPβ**, CCAAT/enhancer binding protein beta

**CCR7**, C‐C chemokine receptor type 7

**CD**, cluster of differentiation (e.g., CD4, CD8, CD11c)

**cDC**, conventional dendritic cell

**cDC1**, type 1 conventional dendritic cell

**cDC2**, type 2 conventional dendritic cell

**cGAS**, cyclic GMP‐AMP synthase

**CTL**, cytotoxic T lymphocyte

**CXCL, CCL**, chemokines (e.g., CXCL9, CCL5)

**CXCR3, CXCR4**, chemokine receptors

**DAMP**, damage‐associated molecular pattern

**DC**, dendritic cell

**DC3**, dendritic cell type 3

**EV**, extracellular vesicle

**EVIR**, extracellular vesicle internalizing receptor

**FDC**, follicular dendritic cell

**GPX4**, glutathione peroxidase 4

**HIF‐1α**, hypoxia‐inducible factor 1 alpha

**iAPC**, induced antigen‐presenting cell

**ICOS‐L**, inducible T‐cell costimulator ligand

**IDO1**, indoleamine 2,3‐dioxygenase 1

**IFN**, interferon

**IFN‐III**, type III interferon

**IFN‐α/β**, type I interferons

**IL**, interleukin (e.g., IL‐10, IL‐12, IL‐6, IL‐1β)

**ILC3**, type 3 innate lymphoid cell

**ISG** ^+^ **DC**, interferon‐stimulated gene positive dendritic cell

**MCRPC**, metastatic castration‐resistant prostate cancer

**MHC**, major histocompatibility complex

**moDC**, monocyte‐derived dendritic cell

**mregDC**, mature regulatory dendritic cell

**OX40**, costimulatory molecule

**PAMP**, pathogen‐associated molecular pattern

**pDC**, plasmacytoid dendritic cell

**PD‐L1/PD‐L2**, programmed death ligand 1/2

**PRR**, pattern recognition receptor

**RLR**, RIG‐I–like receptor

**sFLT3L**, soluble fms‐like tyrosine kinase 3 ligand

**SOCS1**, suppressor of cytokine signaling 1

**STING**, stimulator of interferon genes

**TAA**, tumor‐associated antigen

**Tcf1**, transcription factor 1

**TCR**, T‐cell receptor

**tDC**, transitional dendritic cell

**tdLN**, tumor‐draining lymph node

**Tfh**, T follicular helper (cells)

**TLR**, toll‐like receptor

**TME**, tumor microenvironment

**Treg**, regulatory T cell

**TriMix**, mix of mRNAs (CD70, CD40L, caTLR4)

**VEGF**, vascular endothelial growth factor

## The pivotal role of dendritic cells in tumor surveillance

The immune system plays a crucial role in recognizing and eliminating nascent tumor cells—a process broadly referred to as cancer immunosurveillance. This multistep mechanism includes immune detection, cytotoxic effector activation, and the clearance of transformed cells. Central to this immune orchestration are dendritic cells (DCs), which act as professional antigen‐presenting cells (APCs) and vital sentinels of tissue integrity. Through their unique ability to connect innate and adaptive immunity, DCs serve as gatekeepers of antitumor immunity by initiating, shaping, and modulating immune responses against tumor antigens.

The tumor immune response unfolds in three interrelated phases: elimination, equilibrium, and escape, collectively known as the cancer immunoediting process [1]. During the elimination phase, the innate immune system—composed of natural killer (NK) cells, macrophages, and DCs—detects transformed cells through stress ligands and danger signals. DCs, in particular, internalize tumor‐associated antigens (TAAs) released from dying cells or exosomes, process them, and migrate to tumor‐draining lymph nodes (tdLNs) to present these antigens to the immune system [2, 3]. In the equilibrium phase, DCs assist in maintaining immunological control over tumor outgrowth by continuously activating T cells. However, tumors may eventually enter the escape phase, characterized by the suppression or evasion of immune responses, often by subverting the functions of DCs through immunosuppressive cytokines (e.g., IL‐10, TGF‐β) or metabolic constraints within the tumor microenvironment (TME) [2, 4].

This review examines how DCs contribute to tumor control, the challenges associated with their functional impairment in the TME, and the latest advancements in DC‐based therapeutic strategies, connecting recent progress in fundamental DC biology with translational approaches for next‐generation cancer immunotherapies.

### The ontogeny of dendritic cells

First identified by Ralph Steinman and Zanvil Cohn in the 1970s, DCs have since been recognized for their unique ability to capture, process, and present antigens to naïve T cells, bridging innate and adaptive immunity [5]. In addition to their role in immune activation, DCs are essential for maintaining immune tolerance, preventing autoimmunity by inducing regulatory T cells and anergic states in self‐reactive lymphocytes.

**Fig. 1:**
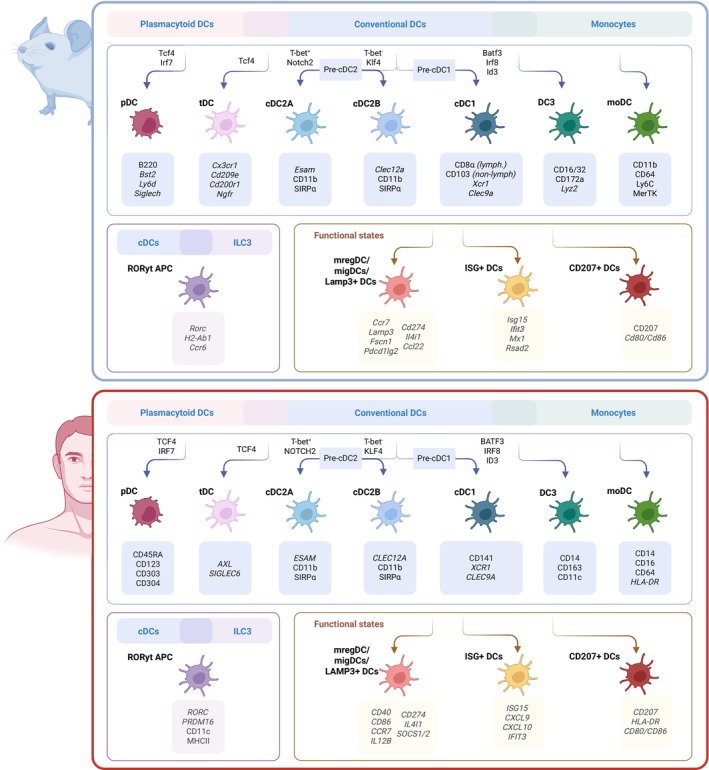
Lineage and functional diversity of dendritic cells (DCs) and related populations. This schematic depicts developmental trajectories and phenotypic markers of murine (top) and human (bottom) plasmacytoid DCs (pDCs), conventional DC subsets (cDC1, cDC2A/B), DC3, monocyte‐derived DCs (moDCs), and transitional DCs (tDCs). Key transcription factors and surface markers are shown for each subset. Additional panels highlight specialized subsets, including RORγt^+^ APCs with shared features of cDCs and ILC3s, and DCs in distinct functional states such as mregDCs (LAMP3^+^ regulatory DCs), ISG^+^ DCs (interferon‐stimulated gene–expressing), and CD207^+^ DCs. These subsets contribute to context‐specific immune responses and reflect the phenotypic plasticity of the DC compartment. Created in BioRender. https://BioRender.com/hgnv6x1.

The DC compartment is heterogeneous, encompassing several major subsets with distinct ontogenies and functions (Fig. 1). DCs in both humans and mice originate from hematopoietic stem cells (HSCs) in the bone marrow through a series of hierarchically organized progenitor stages. The differentiation trajectory begins with the common myeloid progenitor (CMP), which gives rise to the macrophage‐dendritic cell progenitor (MDP). An MDP subsequently differentiates into the common dendritic cell progenitor (CDP), a central node from which both plasmacytoid DCs (pDCs) and conventional DCs (cDCs) derive [2, 6, 7]. From the CDP, lineage commitment proceeds through two primary precursor populations: pre‐pDCs and pre‐cDCs. Pre‐pDCs give rise to the pDCs lineage, characterized by their strong production of type I interferons (IFN‐α/β) in response to viral nucleic acids detected by endosomal TLR7 and TLR9. In mice, pDCs express Bst2, Ly6D, and SiglecH, relying on transcription factors such as TCF4 and IRF7 for their development [8]. In humans, pDCs express CD123 (IL‐3Rα), CD303 (BDCA‐2), and CD304 (BDCA‐4/Neuropilin‐1) and share the same transcription factor dependencies [9]. Although their antiviral role is well‐established, pDCs also demonstrate context‐dependent immunosuppressive functions in the tumor microenvironment and chronic infections, where they can contribute to immune evasion by promoting regulatory T‐cell induction [10].

Meanwhile, pre‐cDCs exit the bone marrow and migrate to peripheral tissues, where they differentiate into type 1 and type 2 conventional DC (cDC1 and cDC2) subsets under the influence of local cytokines and environmental cues [11]. Murine cDC1s are characterized by the expression of CD8α (in lymphoid tissues), CD103 (in nonlymphoid tissues), XCR1, CLEC9A, and elevated levels of MHC class I (MHCI) and costimulatory molecules. Their development relies on transcription factors such as BATF3, IRF8, and ID2 [7, 12, 13]. Human cDC1s express CD141 (BDCA‐3), XCR1, and CLEC9A, relying on BATF3 and IRF8 as well. Functionally, cDC1s are uniquely equipped for cross‐presentation of exogenous antigens on MHCI molecules and play a nonredundant role in activating cytotoxic CD8^+^ T lymphocytes (CTLs), particularly in the context of viral infections and cancer [13, 14]. Murine cDC2s express CD11b and SIRPα and rely on the IRF4, KLF4, and Notch2 signaling pathways for differentiation. Human cDC2s are typically defined by CD1c (BDCA‐1) and SIRPα expression, with similar transcriptional requirements (IRF4, Notch2) [15]. Functionally, cDC2s are crucial for priming naïve CD4^+^ T cells, guiding their polarization into Th1, Th2, Th17, or T follicular helper (Tfh) subsets, depending on contextual signals [11, 16]. This plasticity is further shaped by tissue‐derived cues such as retinoic acid, TGF‐β, and lymphotoxins, which influence cDC2 subset specification and function.

Recent findings challenge the notion that DC subset specialization occurs exclusively in peripheral tissues. Instead, ontogenetic imprinting begins in the bone marrow. In particular, the murine cDC2 lineage has been shown to bifurcate early into two transcriptionally and functionally distinct branches: T‐bet^+^ cDC2A and T‐bet^−^ cDC2B. These subsets differ in their transcription factor dependencies—that is, Notch2 for cDC2As and KLF4 for cDC2Bs—and also exhibit distinct precursor identities at the pre‐cDC2 level [11, 15]. For instance, SiglecH^+^ pre‐cDC2A cells preferentially give rise to T‐bet^+^ Esam^+^ cDC2As, while SiglecH^−^ pre‐cDC2Bs generate CLEC12A^+^ cDC2Bs, suggesting that cDC2 heterogeneity is rooted in ontogenetic divergence rather than solely in peripheral plasticity. cDC2As are more pro‐inflammatory, supporting Th1 polarization, and often originate from Notch2‐dependent ESAM^+^ precursors. In contrast, cDC2Bs, which are CLEC12A^+^ and KLF4‐dependent, favor Th2 and regulatory responses. The classification of cDC2A and cDC2B subsets in humans is still evolving, but recent single‐cell and transcriptomic studies suggest that functionally and transcriptionally analogous populations to murine cDC2A and cDC2B exist in humans [9].

In parallel, an additional DC subset—transitional DCs (tDCs)—has been identified that blurs the boundary between pDCs and cDC2s [17, 18]. In mice, these tDCs arise from bone marrow progenitors that share features with the pDC lineage but can differentiate into ESAM^+^ DC2s while retaining pDC‐like transcriptional traits. Their ontogeny is TCF4‐dependent, and fate‐mapping studies confirm their shared ancestry with pDCs. However, tDCs exhibit unique pro‐inflammatory functions, including IL‐1β secretion during viral infections, a property not shared by canonical pDCs. In humans, tDCs can be distinguished from pDCs by their expression of AXL, while surface or intracellular Axl protein is undetectable in murine tDCs, suggesting that this marker is not conserved across species [18]. Single‐cell RNA sequencing (scRNAseq) studies have identified two main subsets of tDCs based on CD11c expression: CD11c low tDCs, which closely resemble pDCs, and CD11c high tDCs, which exhibit features of cDCs. CD11c low tDCs express high levels of CD69, a marker of lymphocyte activation, while CD11c high tDCs upregulate MHC class II and CD86. In both mice and humans, tDCs exhibit limited production of type I interferons (e.g., IFN‐α) and IL‐12p70 compared to pDCs, although they do produce the precursor IL‐12p40 upon stimulation with CpG‐A or CpG‐B.

Moreover, monocyte‐derived dendritic cells (moDCs) and the more recently described DC3s introduce further layers of developmental complexity. moDCs arise during inflammation from classical monocytes (Ly6C^+^ and CD14^+^, respectively, in mice and humans) and rapidly populate peripheral tissues, often acquiring antigen‐presenting and immunostimulatory functions in response to pathogens or tissue damage. In contrast, DC3s originate from monocyte‐dendritic cell progenitors (MDPs) and are both phenotypically and transcriptionally distinct from cDCs and monocytes. DC3s, recently described in humans, are characterized by the expression of monocyte‐associated markers (CD14, CD163), along with DC‐defining features such as HLA‐DR and CD11c [19, 20]. Their identification across species, including humans, along with their roles in cancer and infection, highlights their significance as a bona fide DC lineage.

Recently, a novel population of APCs expressing the transcription factor RORγt has been identified and proposed as a distinct lineage. These RORγt^+^ APCs are characterized by a hybrid transcriptional program, combining features of cDCs with tolerogenic molecules such as PD‐L1, IL‐10R, and PRDM1. Functionally, they exhibit CCR7‐dependent migration, reside in multiple tissues (including lymph nodes and mucosa), and can prime naïve CD4^+^ T cells—all key hallmarks of professional APCs. Their identity is distinct from ILC3s and cDCs, and they are conserved across murine and human systems [16, 21]. While they have not been studied in tumor contexts yet, their properties suggest potential relevance in settings of immune regulation and tolerance.

Ontogeny is not just a framework for classification; it deeply influences DC function. The transcriptional programs initiated in the bone marrow shape the subsequent responsiveness of DCs to tissue cues, ultimately determining their ability to support immunity or tolerance. This carries practical implications: manipulating early differentiation pathways, such as FLT3L‐driven expansion or Notch/KLF4 axis modulation, could enhance specific DC outputs for therapeutic benefits in cancer or infectious disease.

### Functional states of dendritic cells

The functional specialization of DC subsets is closely linked to their ontogeny, transcriptional programming, and the environmental context in which they operate. Although they serve as APCs, distinct DC subsets vary significantly in their capacity to sense, process, and present antigens, as well as in their ability to orchestrate specific T‐cell responses, regulate tolerance, and shape the immune microenvironment. Beyond their ontogeny, DCs exhibit remarkable functional plasticity shaped by environmental cues, maturation signals, and tissue‐specific microenvironments. This plasticity allows DCs to assume various functional states that go beyond traditional lineage classification, often bearing significant implications for immunity, tolerance, and disease progression. The maturation of DCs is a dynamic, multistage process that enables the transition from peripheral antigen‐capturing cells to migratory, T cell–priming APCs. In a steady state, immature DCs continuously sample their environment with low levels of costimulatory molecules. Upon encountering pathogen‐associated molecular patterns (PAMPs) or damage‐associated molecular patterns (DAMPs), they undergo phenotypic and functional maturation, marked by the upregulation of MHCII, CD80, CD86, and CCR7, as well as increased cytokine production [2]. However, maturation is not a binary state. Recent single‐cell transcriptomic studies have revealed multiple coexisting mature DC states, shaped by environmental conditions, epigenetic programming, and cellular metabolism.

One of the most prominent context‐dependent DC states is the mature DC enriched in regulatory molecules (*mregDC*). This state is characterized by the co‐expression of canonical maturation markers (CD40, CD86, CCR7, and IL12B) and immunosuppressive molecules such as PD‐L1, PD‐L2, IL4I1, SOCS1, and IDO1 [9, 22]. In mice, mregDCs develop from cDC1 and cDC2 lineages upon exposure to environmental signals such as tumor‐derived factors or cell debris rich in cholesterol. In humans, they are derived from CD141^+^ cDC1s and CD1c^+^ cDC2s, and express PD‐L1, PD‐L2, IL4I1, and CMTM6, among others [22]. Functionally, mregDCs retain their ability to activate T cells; however, they also restrict excessive immune activation and promote peripheral tolerance. Within the TME, mregDCs may suppress effector T‐cell responses and contribute to immune evasion. These dual characteristics emphasize the potential of mregDCs as both obstacles and targets in cancer immunotherapy. Notably, mregDC formation has been linked to the uptake of apoptotic tumor debris and the reprogramming of lipid metabolism.

Both cDC1 and cDC2 can give rise to LAMP3^+^ DCs, a terminally differentiated and immunoregulatory subset whose transcriptomic state is primarily developmentally determined rather than cancer‐dependent [23]. These cells exhibit an activated phenotype and high migratory capacity [24]. Unlike conventional DCs, LAMP3^+^ DCs display impaired pro‐inflammatory pathways, as evidenced by reduced TLR signaling, while expressing high levels of immunosuppressive markers (PD‐L1 and PD‐L2) and chemokines (CCL22 and CCL19) that recruit Tregs and promote their differentiation. In humans, LAMP3^+^ DCs often co‐express CMTM6, a key regulator of PD‐L1 stability [25], further reinforcing their role in immune evasion. The immunosuppressive axis formed by LAMP3^+^ DCs in conjunction with exhausted CD8 T cells and Tregs has been consistently observed across multiple studies, creating localized immune evasion “hotspots” within the TME [26, 27, 28]. In human colorectal and nasopharyngeal cancer, LAMP3^+^ DCs show distinct lineage‐specific profiles depending on their origin from cDC1 or cDC2, with corresponding chemokine expression patterns (CCL22 vs CCL17) and immunomodulatory interactions [29].

Extending the current understanding of immunoregulatory DC subsets, RORγt^+^ APCs have been shown to play a central role in oral tolerance and Treg induction in mucosal tissues. Studies in intestinal environments have demonstrated that PRDM16‐dependent RORγt^+^ DCs, as well as extrathymic Aire‐expressing cells (eTACs) and Thetis cells, promote the expansion of Foxp3^+^ peripheral Tregs in response to dietary antigens [30, 31]. Their expression of immune checkpoint ligands, migratory behavior, and conserved tolerogenic signature suggest that analogous populations may emerge in tumors, particularly those arising at mucosal or epithelial sites. Although their role in cancer remains unexplored, these APCs expand the conceptual framework of DC plasticity and peripheral immune regulation.

Another functionally distinct state is represented by ISG^+^ DCs, which upregulate genes associated with the type I interferon response, including *ISG15*, *CXCL9*, *CXCL10*, and *IFIT3* [32]. These DCs have been identified in both steady‐state tissues and inflamed environments, such as tumors, where they play roles in antiviral defense and potentially in the cross‐priming of CD8^+^ T cells. In murine models, ISG^+^ DCs may serve as an early maturation intermediate before transitioning to the mregDC state, although this transition remains incompletely understood. In humans, ISG^+^ DCs have been described in regressing tumors as well. ISG^+^ DCs activate CD8^+^ T *cells ex vivo* comparably to cDC1 and promote antitumor immunity without cDC1s. Unlike cross‐presenting DCs, ISG^+^ DCs acquire and present intact tumor‐derived peptides on MHCI complexes [32].

Tissue‐specific factors also imprint distinct DC states. For example, CD207^+^ DCs, which resemble Langerhans cells, have been identified in several human tumors and peripheral tissues. These cells originate from cDC2s under the influence of TGF‐β and retinoic acid, expressing *CD1A* and *FCGBP*. They are linked to favorable outcomes in certain cancers, such as breast and colorectal carcinomas [33]. In mice, CD207 identifies both self‐renewing Langerhans cells and a subset of CD103^+^ cross‐presenting cDC1s in nonlymphoid tissues. While Langerhans cells originate from embryonic precursors, CD207^+^ cDC1s arise from FLT3L‐dependent progenitors and are specialized in antigen cross‐presentation, particularly in the context of viral infections and tumor immunity [34].

## Dendritic cells as key players in the “cancer‐immunity cycle”

Given their ability to prime and expand antigen‐specific CD4^+^ and CD8^+^ T‐cell responses, DCs are crucial players in the so‐called “cancer‐immunity cycle,” a series of stepwise events that generate anticancer immune responses [35]. In particular, cDC1s serve as vital initiators of CD8^+^ T‐dependent antitumor immunity. After capturing neoantigens, cDC1s process them and migrate to tdLNs to cross‐present tumor antigens on MHCI, stimulating naïve CD8^+^ T cells. Once activated, effector T cells migrate to and infiltrate the tumor, recognizing and binding to cancer cells via T‐cell receptor (TCR)‐MHCI interactions, leading to cancer cell lysis. This process releases additional TAAs, fueling subsequent cycles of immune activation and strengthening the response over time. Progress in the field of cancer immunity has suggested the existence of a cancer‐immunity subcycle within the TME. In this immunologic microcosm, infiltrating T cells move through the stroma and interact with immune cells, including DCs, that are interspersed or aggregated in the tumor parenchyma or tertiary lymphoid structures. T cells then expand and differentiate locally, leading to direct tumor cell killing [36].

### Contributions of dendritic cell subsets to antitumor immune responses

Within the TME, cDC1s include at least three functionally distinct subpopulations that coordinate antitumor immunity: immature cDC1s, characterized by low MHCII expression and located within the tumor parenchyma; activated, nonmigratory cDC1s, which display high MHCII expression and lack CCR7, participating in the organization of stromal CD8^+^ T cell niches by secreting CXCL9; and migratory, immunoregulatory cDC1s that also express high levels of MHCII but are CCR7‐positive. Analysis of spatial transcriptomics using deep learning techniques has revealed that clustered arrangements of CXCL9+ cDC1s and stem‐like TCF1^+^ CD8^+^ T cells at the tumor–stroma interface correlate with tumor regression [6]. Moreover, tumor‐bearing mice lacking the CXCL9 receptor CXCR3 show poor responses to anti‐PD1 treatment, suggesting that CXCL9 production by cDC1s is essential for facilitating the tumor immune response.

Analyses of tumor mouse models did not assign predictive value to CCR7 expression in intratumoral cDC1, suggesting that CCR7^+^ cDC1 may not be involved in forming clusters with CD8^+^ T cells. Instead, these cells were predominantly located in the tumor's peripheral (stromal) regions, which might be due to IDO1 expression [6]. CCR7^+^ cDC1 cultures selectively expressed the IDO1 enzyme, both at steady state and following stimulation with LPS. These CCR7^+^IDO1^+^ cDC1 were shown to suppress T‐cell responses—evidenced by reduced proliferation of ovalbumin‐specific CD8 (OT‐I) T cells and lower IFN‐γ production—and to promote the expression of the immune checkpoint molecules PD‐L1 and PD‐L2. Consistent with their immunoregulatory phenotype, L‐kynurenine—a metabolite of tryptophan catabolism mediated by IDO1—was detected in wild‐type CCR7^+^ cDC1s but not in *Ido1* ^−/−^ cDC1. Furthermore, cDC1‐derived L‐kynurenine induced IDO1 expression in cDC2s via activation of the aryl hydrocarbon receptor (AhR), thereby conferring regulatory properties to cDC2 [37].

While cDC1s drive antitumor immunity, many tumors favor cDC2 dominance, often creating an immunosuppressive environment that hinders effective immune responses [23]. The role of cDC2 in tumor immune responses is less defined than that of other DCs, likely due to their heterogeneity and the lack of unique markers for identification. After capturing and processing antigens, cDC2s migrate to the tdLNs, transferring antigens to resident DCs or directly priming CD4^+^ T cells [33]. Indeed, cDC2s are crucial promoters of CD4^+^ T helper cell responses via the presentation of MHCII‐associated tumor antigens. Two distinct IRF4‐dependent CD11b^+^ cDC2 subsets have been identified in both murine and human tdLNs and TMEs [38]. Among these, the migratory CD11b^+^ CD301b^−^ subset appears to be more immunostimulatory, particularly following Treg depletion, which restores their chemokine and costimulatory profiles and enhances their ability to initiate Th1‐like CD4^+^ T‐cell responses. The cDC2:Treg ratio correlates with CD4^+^ T‐cell infiltration in the TME, with low cDC2 numbers associated with reduced CD4^+^ priming, suggesting that Tregs limit cDC2 licensing. In human head and neck squamous cell carcinoma, a strong correlation is observed between BDCA‐1^+^ cDC2 abundance, Treg levels, and CD4^+^ T‐cell phenotypes, supporting the translatability of murine cDC2 findings to human tumors. Importantly, BDCA‐1^+^ cDC2 levels may predict responses to PD‐1 blockade therapy, even in tumors with low BDCA‐3^+^ cDC1 content, particularly when tumor cells express MHCII and engage CD4^+^ T cells [38]. Analysis of two large human cancer datasets revealed that the prognostic value of DC subsets is highly context‐dependent. In T‐cell‐resistant tumors (low CD8 T cell and high M2‐like macrophage levels), cDC2 infiltration correlates more strongly with patient survival than cDC1 [39], indicating that the cDC2‐CD4 T‐cell axis is a potent alternative route for inducing antitumor immunity, especially in tumors resistant to CTL responses.

Among cDC2 subsets, CXCL9^+^ cDC2s were predicted to be a major precursor of LAMP3^+^ cDCs. During the transition from CXCL9^+^ cDC2s to LAMP3^+^ cDCs, CXCL9 expression decreases while IDO1 expression increases [23]. LAMP3^+^ DCs are involved in tryptophan metabolism via IDO1 and can also promote pro‐angiogenic signaling through the VEGFA–NRP2 axis, contributing to tumor progression [27].

pDCs also play a crucial role in shaping the TME, partly due to their increased sensitivity to type III interferon (IFN‐III). Among immune cell populations, pDCs—both in circulation and within tumors—demonstrate the highest levels of IFNLR1, which facilitates robust activation of the STAT1 pathway upon IFN‐III stimulation. This activation improves their immunomodulatory profile by upregulating PD‐L1 and ICOS‐L and prepares them for stronger type I interferon (IFN‐I) responses, especially following TLR7 engagement [10].

Functionally, tDCs demonstrate an intermediate immunostimulatory capacity: CD11c low tDCs behave similar to pDCs, whereas CD11c high tDCs are more efficient at antigen presentation and T‐cell activation, akin to cDCs. This dual identity, conserved across species, highlights the role of tDCs as modulators of immune responses within the TME, particularly when cytokine production and T‐cell priming are essential for effective antitumor immunity.

### Recruitment and activation of dendritic cells within the tumor microenvironment

The accumulation of DCs within the TME is regulated by local growth factors and chemokines that promote recruitment, differentiation, and expansion. NK cells play a crucial role by producing FLT3L, which supports the differentiation of cDC precursors and the expansion of cDC1s. Additionally, NK cells secrete XCL1 and CCL5, which specifically attract cDC1s, underscoring an NK‐DC interaction that enhances tumor immune surveillance [40]. Tumor‐derived CCL4 also facilitates the recruitment of cDC1s. The presence of NK cells in the TME correlates with higher levels of cDC1s and an improved response to anti‐PD‐1 immunotherapy in melanoma patients [41].

The simultaneous release of tumor antigens and DAMPs significantly enhances the ability of DCs to capture, process, and present TAAs. cDC1s have a variety of specialized receptors that efficiently internalize apoptotic bodies and necrotic cell debris, primarily through phagocytosis. Among these, C‐type lectin receptors (CLRs) on DCs play crucial roles in recognizing and internalizing tumor antigens for cross‐presentation. For instance, CLEC9A is exclusively expressed by cDC1 and detects exposed polymerized F‐actin, signaling via SYK [42]. CLEC9A quickly internalizes and directs antigens into the cross‐presentation pathway, although tumor‐secreted gelsolin can impair its function [43].

After antigen capture, DCs undergo a tightly regulated process of maturation (or immunogenic activation), during which cDCs transition from being primarily antigen‐capturing cells to becoming highly efficient at antigen presentation, T‐cell activation, and tissue migration. Maturation is characterized by the downregulation of endocytic and antigen‐processing machinery, along with the upregulation of MHCI/II, costimulatory molecules (CD80, CD86, and CD40), and the chemokine receptor CCR7, facilitating interaction with T cells and migration to lymphoid organs. Activated cDC1s also modify their cytokine secretion profile to enhance pro‐inflammatory immune responses, including the production of IL‐12 and type I/III interferons. cDC1 activation is initiated by PAMPs or DAMPs through pattern recognition receptors (PRRs) such as TLRs, CLRs, RIG‐I‐like receptors (RLRs), and the cytosolic DNA sensor cGAS [44]. Importantly, cDC1 subsets—including CD8α^+^ and CD103^+^ DCs—express high levels of TLR3, which allows them to detect double‐stranded RNA in endosomes and respond by activating TRIF‐dependent pathways. This triggers the induction of type III interferons and enhances DC immunostimulatory capacity [45]. Moreover, when tumor cells undergo necrosis or stress, they release dsDNA, which can be absorbed by cDC1s and recognized by cGAS. This activates the STING pathway via cGAMP, leading to TBK1 activation, nuclear translocation of NF‐κB and IRF3, and the transcription of immune effector genes, including IFNβ, IL‐12, IL‐6, TNF, and CXCL9, as well as costimulatory molecules [46].

Following activation and maturation in the TME, cDC1s enter a migratory state that enables them to travel to tdLNs and engage cognate naïve T cells in lymph nodes. Studies using intravital imaging have identified migratory CD103^+^ cDC1 in mice (BDCA‐3^+^ cDC1 in humans) as the key subset responsible for transporting tumor antigens to the tdLN for the cross‐priming of naïve CD8^+^ T cells. Following maturation, CCR7^+^ cDC1s migrate to tdLNs to prime naïve T cells, while CCR7^−^ cDC1s act locally, as outlined in the previous section.

### Licensing, cross‐presentation, and cross‐priming of CD8 ^+^ T cells

The efficiency of antigen capture, processing, and presentation by APCs affects the type of T‐cell response against cancers. Among APCs, cDC1 is a specialized subset responsible for tumor antigen cross‐presentation and the cross‐priming of CD8^+^ T cells. Cross‐presentation involves the presentation of exogenous antigens by APCs as peptides associated with MHCI molecules, while cross‐priming is the recognition of the peptide‐MHCI complex by CD8^+^ T cells, leading to clonal expansion and activation of cytotoxic responses [47, 48]. Notably, mice that lack the cDC1 compartment, or mice genetically deficient in the expression of β2 microglobulin of MHCI specifically in cDC1s, when challenged with tumors, fail to induce antigen‐specific CD8^+^ T‐cell responses and to reject tumors [14].

Following internalization, tumor‐derived antigens may be processed by lysosomal proteases and loaded onto MHCI directly in the endosome (i.e., vacuolar pathway) or exported to the cytosol from the endosome/phagosome, where they are processed by the proteasome into peptides that are loaded onto MHCI (i.e., cytosolic path). The molecular mechanism mediating antigen trafficking from the phagosome to the cytosol is not completely clear; it may involve Sec22b, WDFY4, perforin 2, and SYK signaling triggered by CLEC9A [49, 50, 51]. Notably, the relevance of both models has not been supported by *in vivo* genetic evidence yet. In both cases, cross‐presentation by DCs cross‐primes CD8^+^ T cells specifically to MHCI‐restricted antigen.

To achieve an effective response against tumors, CD8^+^ T cells require assistance from CD4^+^ T cells. The general principle indicates that the priming of CD4^+^ T cells is preferentially performed by cDC2s, which capture and present tumor‐derived antigens. However, some evidence contests the necessity of cDC2s in priming CD4^+^ T cells, as cDC1s have been shown to be more efficient than cDC2s in presenting cell‐associated antigen. In the revised model, cDC1s serve as the platform for priming both CD4^+^ and CD8^+^ T cells. The ligation of CD40 expressed by cDC1 during CD4^+^ T‐cell priming is critical for robust licensing of cDC1s; without this interaction, tumor rejection mediated by CD8^+^ T cells may fail [14, 52]. CD40 ligation enhances CD8^+^ responses by inducing the expression of several costimulatory molecules, which increase CD8^+^ T cell activation, such as CD70 and 4‐1BBL, along with antiapoptotic proteins such as BCL‐xL and BCL‐2, providing resistance to FAS‐mediated apoptosis triggered by interactions with activated CTLs or NK cells. In addition to CD40 licensing of cDC1, the production of cytokines and direct activation of CD40 signaling in CD8^+^ T cells have also been proposed [52]. It has been demonstrated that helped and nonhelped CD8^+^ T cells present a distinct gene signature [53]—helped CTLs exhibit upregulation of key effector genes (e.g., granzymes, IFN‐γ), survival markers (e.g., IL‐7R, IL‐2/CD25), and migration‐associated receptors (e.g., CX3CR1, CXCR4), while showing reduced expression of inhibitory receptors such as PD‐1, BTLA, and LAG3. Moreover, CTLs primed in the presence of CD4^+^ T cell help possess enhanced, cell‐intrinsic abilities to traffic to the tumor site and consequently control the tumor.

The interaction between MHCI‐peptide and the TCR provides the primary activation cue, known as Signal 1. However, full activation of CD8^+^ T cells requires two additional inputs. Signal 2 arises from costimulatory molecules expressed on the surface of cDC1s, including CD80, CD86, 4‐1BBL, and CD70. The CD70‐CD27 pathway has emerged as a critical mediator of CD4^+^ T‐cell help, alongside DC‐derived cytokines such as IL‐12 and IL‐15 (Signal 3). This pathway significantly enhances the amplification and persistence of CD8^+^ T‐cell activity within the tumor microenvironment [53]. While cDC1‐mediated cross‐priming of CD8^+^ T cells has traditionally been associated with tdLNs, emerging evidence indicates that this process can also occur directly within the TME [54].

Beyond recruitment, NK cells and cDC1s engage in reciprocal activation within the TME. cDC1s produce IL‐12, activating NK cells, while NK cells secrete IFN‐γ, further stimulating cDC1 function [55]. This bidirectional communication amplifies the antitumor immune response, promoting the activation and infiltration of effector T cells.

A recently described phenomenon termed “cross‐dressing” adds another layer of complexity to the function of DCs [56]. This process involves transferring preformed peptide–MHC (p‐MHC) class I or II molecules from tumor cells to DCs, including both cDC1s and cDC2s, through the uptake of tumor‐derived extracellular vesicles or via horizontal transfer mechanisms such as trogocytosis at the plasma membrane [57, 58]. Cross‐dressing has been observed in both *in vitro* and *in vivo* tumor models and can be amplified through DC engineering for therapeutic purposes. Accordingly, DCs may be equipped with a chimeric receptor called EV‐internalizing receptors (EVIR), which can enhance the uptake of tumor‐derived EVs and membranes, thereby improving DC cross‐dressing [59].

## Impairment of dendritic cell function and antitumor immunity by the tumor microenvironment

**Fig. 2:**
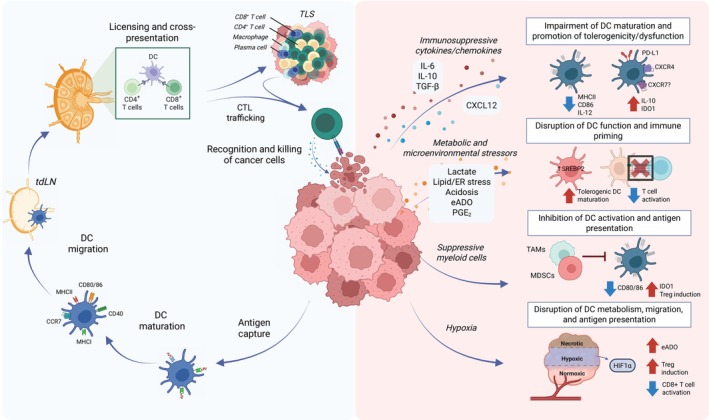
Tumor‐mediated suppression of dendritic cell (DC) function impairs antitumor immunity. The scheme illustrates the normal DC‐mediated cancer immunity cycle (left), including antigen capture, maturation, migration to tumor‐draining lymph nodes (tdLNs), T cell priming, and cytotoxic T lymphocyte (CTL) trafficking. In contrast, the tumor microenvironment (right) disrupts this process through immunosuppressive cytokines (*e.g*., IL‐6, IL‐10, TGF‐β), metabolic stressors (*e.g*., lactate, PGE₂, adenosine), hypoxia, and suppressive myeloid cells. These factors impair DC maturation, antigen presentation, and T cell activation, while promoting tolerogenic phenotypes and regulatory T cell induction. Created in BioRender. https://BioRender.com/422uw16.

Tumor progression has been linked to dynamic changes in the cellular composition of tdLNs, including a progressive decline in APC subsets [60] (Fig. 2). Studies using various orthotopic mouse models of tumors with low T‐cell infiltration and resistance to anti‐PD‐L1 therapy have consistently shown a reduced abundance and impaired functionality of migratory CD103^+^ cDC1 cells, leading to defective T‐cell priming [61]. Restoring their function often requires additional stimulation, such as FLT3L, radiotherapy, or TLR3/CD40 agonists. However, individual interventions are frequently insufficient to fully reverse cDC1 dysfunction, underscoring the need for multifactorial therapeutic strategies [61]. Tumor‐infiltrating cDC1s often display an immature phenotype, as tumor‐intrinsic factors and soluble components of the TME, including cytokines, chemokines, metabolites, and signaling molecules, can suppress their migration to lymph nodes. In the KPC pancreatic cancer model (*LSL‐Kras* ^*G12D/+*^;*LSL‐Trp53* ^*R172H/+*^;*Pdx‐1‐Cre*), high serum IL‐6 levels were associated with reduced cDC1 numbers, which correlated with increased cell death of mature cDC1s [62].Using IL‐6‐expressing EL4 cells and *in vitro* cultures of cDC1 precursors, it was demonstrated that tumor‐derived IL‐6 directly impairs cDC1 differentiation from hematopoietic progenitors by blocking pre‐cDC1 specification through C/EBPβ induction. In mouse and human cord blood–derived cells, IL‐6 caused a dose‐dependent reduction in cDC1 development, while cDC2 differentiation remained largely unaffected [63].

Immunosuppressive signals within the TME further contribute to cDC dysfunction. For example, IL‐10 secretion and reduced IL‐12 production are associated with impaired DC activity. Although IL‐10 did not directly affect CD8^+^ T cells or macrophage polarization, blocking its receptor (IL‐10R) increased IL‐12 expression in DC subsets (CD103^−^CD11b^+^ and CD103^+^CD11b^−^), thereby enhancing antitumor responses [64]. Among non‐T cells, myeloid cells—particularly DCs—exhibited significantly higher IL‐10R expression than neutrophils, B cells, and other immune cell types. Alongside Tregs, DCs play a central role in promoting metastasis [65], making IL‐10R blockade a promising antimetastatic strategy [66]. Supporting this, another study demonstrated that transducing self‐differentiated dendritic cells (SD‐DCs) from human monocytes with shRNAs targeting IL‐10RA and TGF‐βRII enhanced their function and boosted T‐cell activation against cholangiocarcinoma cells [67]. Other immunosuppressive tumor‐derived factors, such as TGF‐β, may also hinder cDC1 antitumor activity by inhibiting antigen uptake and cytokine production.

In the early stages of tumor progression, the presence of cDC1s in the TME is limited due to the preferential recruitment of tumor‐promoting immune cells by chemokines such as C‐X‐C motif chemokine ligand 1 (CXCL1), C‐C motif chemokine ligand 2 (CCL2), and CCL20, which are secreted by tumor cells [68]. As key producers of chemokines, tumor cells attract protumor immune populations and restrict the infiltration of antitumor cDC1s, thereby promoting tumor growth. For instance, CXCL12, secreted by tumor, stromal, and immune cells, binds to CXCR4, facilitating tumor growth, immune evasion, and therapy resistance. Blocking CXCR4 in combination with anti–PD–1 therapy enhances antigen presentation and increases cDC1 infiltration into tumors, as demonstrated in orthotopic and autochthonous hepatocellular carcinoma (HCC) models [69]. Furthermore, CXCR7, another receptor for CXCL12, is expressed on B cells and DCs and modulates immune responses [70], potentially affecting cDC1 function within the TME, which requires further exploration.

Although chemokines such as CCL3, CCL4, CCL5, CCL19, CCL20, CCL21, and XCL1 can contribute to tumor progression by modulating tumor cells and protumorigenic elements of the tumor microenvironment—including fibroblasts, endothelial cells, and immunosuppressive immune cells—they are also recognized for their ability to recruit cDCs and potentially enhance antitumor immunity [71, 72]. Research in murine tumor models has shown that cDC1 infiltration depends on NK cells, which release the chemoattractants CCL5 and XCL1. Similarly, in human tumors, the expression levels of CCL5, XCL1, and XCL2 correlate with the presence of NK cells, cDC1 recruitment, and improved patient outcomes [68]. NK cells are also key producers of FLT3L within the tumor, which regulates cDC1 abundance in the TME and is essential for maintaining their function. Therefore, NK cells serve a dual role in not only attracting cDC1s but also supporting their longevity and functional capabilities [73].

Tumor‐derived prostaglandin E₂ (PGE₂) plays a significant immunosuppressive role by inducing cDC1 dysfunction through EP2/EP4‐mediated cAMP signaling, which downregulates the transcription factor IRF8, critical for cDC1 function and antitumor immunity [74]. The impact of PGE₂ has been reported in earlier studies [75]. In melanoma models lacking COX1/COX2 (*Ptgs1* ^−^/^−^ *Ptgs2* ^−^/^−^), and thus PGE₂, MHCII^hi^CCR7^neg^ cDC1s retain high levels of IL‐12 and CXCL9, allowing them to cluster with tumor‐specific CD8^+^ T cells, promoting their activation and driving local immune control. Additionally, the absence of PGE₂ led to the recruitment of cDC1s into the TME through the chemokines CCL5 and XCL1 [75]. In contrast, in PGE₂‐producing tumors, these cDC1s show reduced CXCL9 expression and fail to support effective T‐cell responses. Blocking PGE₂ signaling restores functional CXCL9^+^CCR7^neg^ cDC1s and improves tumor control, underscoring the role of PGE₂ in disrupting local immune niches [6].

Similarly, tumor‐derived lactate significantly impacts DC dysfunction within the TME by promoting the development of immunosuppressive CD63^+^ mregDCs. As Plebanek et al. (2024) demonstrated [76], these mregDCs are characterized by increased expression of cholesterol biosynthesis genes and depend on the activity of sterol regulatory element–binding protein 2 (SREBP2) for their function. Lactate acts as a metabolic signal that activates SREBP2, reprogramming cDCs into a tolerogenic state that suppresses effective antitumor T‐cell responses. This immunosuppressive transformation can be reversed by genetic deletion or pharmacological inhibition of SREBP2, which restores DC‐mediated immune activation. Additionally, targeting lactate export via MCT1 inhibition may further disrupt this tolerogenic axis, providing a complementary therapeutic strategy to enhance DC function in the TME [76].

In addition to creating an acidic microenvironment, developing tumors generate hypoxic regions that promote the local expression of hypoxia‐inducible factor‐1α (HIF‐1α), reshaping DC function and favoring immune evasion. In HCC, HIF‐1α upregulates CD39 and CD73 in tumor cells, enhancing extracellular adenosine (eADO) production. eADO recruits pDCs via ADORA1, promoting Treg induction and suppressing CD8^+^ T‐cell cytotoxicity, which correlates with poor prognosis [77]. Hypoxic tumor regions are also enriched in immunosuppressive populations, including Tregs, M2 macrophages, and HLA‐DR^low^ cDC2s. Meanwhile, hypoxia‐low areas exhibit a more active immune profile, marked by granzyme B‐expressing CD8^+^ T cells. The interaction between Tregs and cDC2s in hypoxic areas leads to HLA‐DR downregulation on cDC2s, impairing antigen presentation and reinforcing local immune suppression, highlighting Treg–DC cross‐talk as a potential therapeutic target in HCC [78]. In gliomas, HIF‐1α directly drives PD‐L1 transcription under hypoxia, and its inhibition restores DC and CD8^+^ T‐cell activation while enhancing responses to immune checkpoint blockade [79]. Interestingly, in pancreatic tumors lacking HIF‐1α, hypoxia leads to glycogen accumulation and the secretion of IL‐1β and IL‐8, which recruit proangiogenic cDC1 and cDC2 subsets, demonstrating an HIF‐1α‐independent mechanism of immune modulation and tumor progression [80]. These findings underscore the multifaceted impact of hypoxia on immune regulation within the TME and position HIF‐1α and its downstream signaling as valuable therapeutic targets.

The accumulation of immunosuppressive cell populations, such as Tregs, myeloid‐derived suppressor cells (MDSCs), and tumor‐associated macrophages, collectively impairs DC function. Tregs, recruited by tumor‐derived chemokines, suppress DC immunogenicity through multiple mechanisms, including CTLA‐4‐mediated downregulation of CD80/CD86, induction of IDO expression, and secretion of inhibitory cytokines. IDO1 expression within the tumor microenvironment is linked to both immunostimulatory and immunosuppressive effects that impact DC function. While high *IDO1* expression in tumors aligns with an inflamed TME—characterized by increased infiltration of activated CD8^+^ T cells and enhanced responses to the PD‐L1 inhibitor atezolizumab—it is also notably present in follicular dendritic cells (FDCs) within tertiary lymphoid structures (TLS) [81]. Therefore, IDO1 expression in inflamed tumors may contribute to a negative feedback loop, resulting in immunosuppressive effects.

The dynamic regulation of immune cell function within the TME is significantly influenced by signaling pathways that affect DC activity. Disruption of canonical NF‐κB (cNF‐κB) signaling in the TME impairs cDC1 function, which is essential for antitumor immunity. The activation of NF‐κB drives cDC1 maturation and the expression of IFN‐γ‐responsive genes, facilitating CD8^+^ T‐cell activation. In melanoma models, cDC1‐specific knockout of Ikbkb, which is essential for cNF‐κB, hampers DC activation and accelerates tumor growth by diminishing T‐cell responses [2]. Conversely, tumors deficient in oncogenic WNT/β‐catenin signaling, such as the *Braf* ^V600E^ *Pten* ^−/−^ melanoma model, produce CCL4, which supports cDC1 accumulation in the TME [40].

While many studies focus on cDC1 functions in antitumor immunity, cDC2s demonstrate equally crucial yet complex roles. These DCs exhibit remarkable functional versatility, as they can activate cytotoxic T cells through both direct cross‐presentation to CD8^+^ T cells and indirect support via CD4^+^ T helper cells. However, their effectiveness is often undermined in the TME, as evidenced by glioblastoma studies showing cDC2s with diminished HLA‐DR, CD86, and IL‐12 expression [82]. Recent findings in HCC revealed that a marked reduction in cDC2 abundance is particularly severe in aggressive intrahepatic metastasis (IM) tumors compared with multicentric occurrence (MO) lesions and normal tissue [83]. This depletion correlates with IM's immunosuppressive character, marked by Treg dominance and poor clinical outcomes. The clinical impact of cDC2s varies across cancers: while melanoma shows paradoxical associations (both worsened survival and improved outcomes with IL‐12 production), lung cancer demonstrates opposing prognostic values for different cDC2 subsets [84].

Similar to their conventional counterparts, pDCs demonstrate remarkable functional plasticity within the TME, often leading to paradoxical roles in cancer progression. In immunosuppressive TMEs, such as those present in ovarian cancer and HCC, pDCs generally lose their characteristic interferon‐producing ability and instead adopt a tolerogenic phenotype. These dysfunctional pDCs upregulate immune checkpoint molecules like PD‐L1 and IDO1, while also recruiting Tregs, thus facilitating immune escape [85]. Hypoxia and adenosine signaling further amplify this immunosuppressive shift, as seen in HCC, where adenosine receptor activation enhances pDC‐driven Treg expansion and impairs CD8^+^ T cell function [77].

Conversely, in particular breast and colon cancers, pDCs retain their immunostimulatory potential, correlating with improved survival and therapy response [84]. Activated subsets, such as OX40^+^ pDCs in head and neck squamous cell carcinoma, can trigger strong cytotoxic T‐cell responses through TRAIL and granzyme B, demonstrating their ability to counteract tumor progression when properly supported [86]. This duality emphasizes how the TME influences pDC function—either suppressing their effector mechanisms or permitting their antitumor activity.

Among the various TME‐induced changes that shape DC function, metabolic reprogramming has emerged as a critical yet underexplored driver of dysfunction. One significant aspect is the dysregulation of lipid metabolism. Tumor‐derived cytokines promote the accumulation of triacylglycerols, cholesterol esters, and free fatty acids in DCs, leading to the formation of large lipid bodies (LBs) [87, 88]. This lipid overload selectively impairs the cross‐presentation of exogenous antigens while preserving endogenous antigen presentation, thus weakening CD8^+^ T‐cell priming and favoring immune evasion [89].

The accumulation of oxidized lipids in tumor‐associated DCs is often linked to endoplasmic reticulum (ER) stress and is exacerbated by hypoxia and reduced GPX4 expression, which promote ferroptosis‐related stress. These conditions favor the formation of polyunsaturated fatty acid (PUFA)‐rich LBs and enable DCs to absorb peroxidized lipids from the TME, which accumulate on LB surfaces. Early ferroptotic tumor cells further impede DC maturation and cross‐presentation within this altered metabolic environment, while late ferroptotic cells become less immunogenic [89].

In parallel, alterations in cholesterol metabolism and the mevalonate (MVA) pathway also critically influence DC immunocompetence [90, 91]. In the TME, the MVA pathway becomes hyperactive in DCs, producing geranylgeranyl diphosphate (GGPP), a metabolite that activates small GTPases. These small GTPases regulate intracellular antigen trafficking; however, their overactivation accelerates antigen routing to lysosomes, which reduces antigen presentation on MHCI and impairs CTL priming [92]. These lipid‐ and cholesterol‐driven changes in DCs enhance immune tolerance within the TME and contribute to tumor progression.

## Therapeutic strategies targeting dendritic cells

**Fig. 3:**
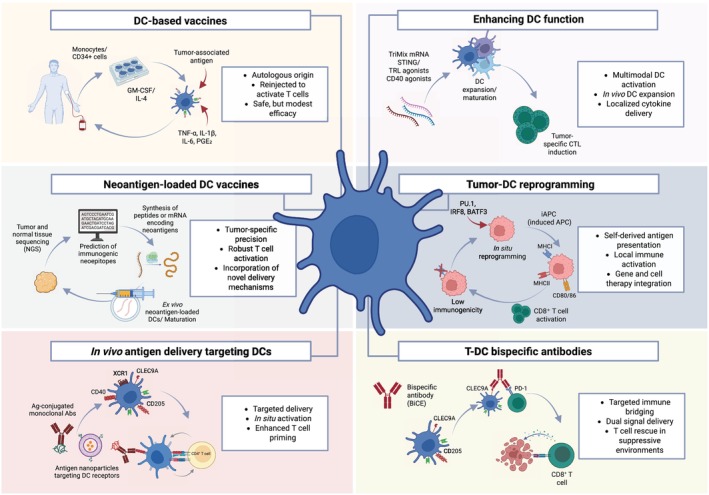
Strategies to harness dendritic cells (DCs) for cancer immunotherapy. This figure summarizes current and emerging approaches to leverage DCs in antitumor immunity. Strategies include: (1) DC‐based vaccines using *ex vivo‐*generated autologous DCs loaded with tumor‐associated antigens; (2) Neoantigen‐loaded DC vaccines providing personalized, mutation‐specific epitopes to enhance T cell responses; (3) *In vivo* antigen delivery targeting DC receptors via antibodies or nanoparticles to improve localized priming; (4) Enhancing DC function through adjuvants and cytokines to boost maturation and CTL induction; (5) Tumor‐DC reprogramming to convert low‐immunogenic tumor cells into functional antigen‐presenting cells; and (6) T‐DC bispecific antibodies to simultaneously engage DCs and T cells, enhancing immune synapse formation and activation even in suppressive tumor microenvironments. Created in BioRender. https://BioRender.com/5xkedbt.

Given their crucial role in initiating and modulating adaptive immune responses, DCs have emerged as attractive targets for therapeutic manipulation in cancer (Table 1). Recent advancements in immunotherapy have resulted in strategies that either enhance DC function, deliver tumor antigens more effectively, or counteract tumor‐mediated suppression of DCs (Fig. 3).

**Table 1:** Comprehensive DC‐based cancer immunotherapy strategies. MCRPC, Metastatic castration‐resistant prostate cancer.

Strategy	Mechanism	Key features	Challenges	Notes	Product name	Active clinical trials	Approval
DC‐based vaccines	*Ex vivo* generation, antigen loading, and reinfusion of autologous DCs	Personalizable; safe; modest efficacy in solid tumors	Labor‐intensive; limited in poorly immunogenic tumors	Optimization with neoantigens, adjuvants, nanoparticle delivery	Sipuleucel‐T (Provenge)	NCT05806814 (Advanced Prostate Cancer) NCT06100705 (MCRPC) NCT05751941 (metastatic Prostate Cancer)	FDA‐approved (MCRPC)
Neoantigen‐loaded DC vaccines	DCs pulsed with patient‐specific mutated peptides or mRNA encoding tumor antigens, neoantigens	Highly specific; robust CD4^+^/CD8^+^ T cell responses	Requires sequencing and epitope validation	Effective in melanoma, glioblastoma; synergizes with checkpoint inhibitors	NeoVax	NCT03422094 (glioblastoma) NCT02950766 (renal cell carcinoma) NCT04930783 (melanoma) NCT04024878 (ovarian cancer)	Investigational
*In vivo* antigen delivery	Antibodies or ligands targeting DEC205, CLEC9A, XCR1 to deliver tumor antigens	Bypasses *ex vivo* handling; Efficient cross‐presentation; CD8^+^ T cell priming	Requires adjuvants; risk of tolerance induction	Used with TLR or CD40 agonists	Antigen‐conjugates (e.g., DEC205‐NY‐ESO‐1)	No currently active trials known	Investigational
Enhancing DC function	Cytokines STING/FLT3L agonists TLR ligands	Expansion of cDC populations; Boosts cDC activation and migration	Systemic toxicity; limited efficacy in aggressive tumors; requires localized delivery	Hydrogels that release cytokines; oncolytic viruses to express cytokines *in situ*	FLT3L; poly(I:C); TriMixDC‐MEL	NCT03789097 (solid tumors) NCT04616248 (metastatic solid tumors) NCT05029999 (breast cancer)	Investigational
Cellular reprogramming	Reprogram tumor cells into DC‐like APCs using PU.1, IRF8, BATF3	Local antigen presentation; self‐sustaining immunity; circumvents DC isolation; generates APCs *in situ*	Delivery and safety of transcription factors	Preclinical proof of concept	None (experimental)	Preclinical	Preclinical
Bispecific T–DC engagers	Bispecific antibodies linking cDC1s to T cells	Enhances cross‐priming; promotes direct DC–T cell synapse formation	Design complexity; requires subset‐specific targeting	Optimize half‐life and tissue distribution	CLEC9A–PD1; CD40–DEC205; PD‐L1 × CD3ε bispecific constructs	Preclinical	Preclinical

### Dendritic cell‐based vaccines

DC‐based vaccines involve the *ex vivo* differentiation, maturation, and antigen loading of autologous DCs, which are subsequently reintroduced into the patient to elicit robust T‐cell‐mediated antitumor responses. Typically, peripheral blood monocytes or CD34^+^ progenitors are isolated and cultured with cytokines such as GM‐CSF and IL‐4 to generate immature DCs. These cells are then exposed to TAAs and maturation stimuli, usually a combination of TNF‐α, IL‐6, IL‐1β, and PGE₂, to develop a mature, immunostimulatory phenotype. Once matured and loaded with antigen, these DCs are reinfused intradermally, subcutaneously, or intravenously, where they migrate to lymph nodes and present tumor‐derived peptides on MHC molecules to naïve CD8^+^ and CD4^+^ T cells. This process results in the generation of CTLs capable of recognizing and killing tumor cells [93].

While DC‐based vaccines have demonstrated excellent safety profiles and the ability to induce tumor‐specific immune responses, their clinical efficacy has been modest, especially in advanced and poorly immunogenic solid tumors such as glioblastoma, pancreatic cancer, and non‐small cell lung cancer. One of the most well‐known examples, Sipuleucel‐T, an FDA‐approved autologous DC‐based vaccine for metastatic prostate cancer, extended overall survival by only 4.1 months in pivotal trials, highlighting the need for more potent formulations [94]. To overcome these limitations, several optimizations are actively being investigated, including (i) antigen selection and loading strategies, ranging from defined tumor peptides and long synthetic peptides to whole tumor lysates and neoantigen‐encoding mRNA, which offer broader epitope coverage and personalized targeting; (ii) next‐generation adjuvants and maturation protocols to drive differentiation of type 1‐polarized DCs (DC1s), characterized by high IL‐12 production and enhanced CTL priming capacity; and (iii) improved delivery routes, such as direct injection into lymph nodes or intratumoral administration, to enhance DC migration and local T‐cell activation. Nanoparticle‐based delivery systems can facilitate *in vivo* targeting of antigens and bring maturation signals to endogenous DCs, potentially replicating the efficacy of *ex vivo* approaches with reduced logistical complexity. Moreover, combination strategies—like DC vaccines co‐administered with immune checkpoint inhibitors or TLR agonists—have shown promising results in preclinical models and early‐phase clinical trials, overcoming the immunosuppressive tumor microenvironment and sustaining T‐cell effector function [95].

### Personalized neoantigen‐loaded dendritic cell vaccines

Personalized neoantigen vaccines represent a cutting‐edge approach in cancer immunotherapy, aimed at overcoming the limitations of shared tumor antigens by targeting patient‐specific mutations. These vaccines involve identifying tumor‐exclusive somatic mutations through next‐generation sequencing of tumor and healthy tissue, followed by the computational prediction of immunogenic neoepitopes—peptides derived from mutated tumor proteins absent in normal tissues. The selected neoantigens are then synthesized as peptides or encoded in mRNA and loaded onto autologous DCs *ex vivo*, which are matured and re‐infused into the patient. This strategy leverages the immunogenic potential of neoantigens, which by definition evade central tolerance mechanisms and can prime high‐affinity CD8^+^ and CD4^+^ T‐cell responses.

Compared with conventional TAA‐based vaccines, neoantigen‐loaded DCs provide increased specificity and a lower risk of autoimmunity. Clinical trials in cancers such as melanoma, glioblastoma, and non‐small‐cell lung cancer have demonstrated that neoantigen‐pulsed DCs can elicit broad and polyfunctional T‐cell responses, promote tumor‐infiltrating lymphocyte (TIL) expansion, and induce clonal replacement of the T‐cell repertoire *in vivo* [96]. In glioblastoma, where immune evasion and low tumor mutational burden pose significant challenges, neoantigen‐loaded DCs have successfully elicited durable responses when combined with checkpoint inhibitors or radiotherapy. Ongoing studies are refining the prediction and validation of immunogenic neoepitopes, optimizing antigen formulation (e.g., long peptides vs. mRNA), and integrating neoantigen DC vaccines with immune checkpoint blockade or TLR agonists to enhance T‐cell priming and persistence. Delivering tumor antigen‐encoding mRNA via lipid nanoparticles to DCs represents a cutting‐edge strategy in cancer immunotherapy [97]. In preclinical models, lipoplexed mRNA vaccines effectively induced tumor rejection and expanded antigen‐specific T cells. Early‐phase clinical data also indicate promising immunogenicity and safety. Notably, mRNA‐4157, a vaccine encoding up to 34 personalized neoantigens, has extended recurrence‐free survival when combined with pembrolizumab in patients with resected high‐risk melanoma [98]. Similarly, autogene cevumeran, another personalized mRNA vaccine encoding 20 neoantigens, demonstrated immunogenicity and relapse prevention signals in surgically treated pancreatic cancer combined with atezolizumab [99]. In summary, mRNA‐nanoparticle vaccines targeting DCs offer a promising platform for personalized cancer immunotherapy, especially when integrated with immune checkpoint blockade.

### *In vivo* antigen delivery strategies targeting dendritic cells

To overcome the logistical and clinical challenges of *ex vivo*‐generated DC vaccines, such as high production costs, inconsistent quality, and limited scalability, *in vivo* antigen delivery methods have been developed to leverage the functionality of endogenous DCs directly within the patient. These strategies aim to target tumor antigens and immunostimulatory signals to specific DC subsets *in situ*, initiating robust T‐cell‐mediated immunity without the need for DC manipulation outside the body. Among the most promising approaches is the use of monoclonal antibodies conjugated with antigens or antigen‐bearing nanoparticles that target DC‐specific surface receptors. For example, CLEC9A, a C‐type lectin receptor selectively expressed on cDC1s, has been used as a delivery route for tumor antigens. CLEC9A‐mediated targeting enhances antigen cross‐presentation via MHCI pathways and significantly improves CD8^+^ CTL priming in both preclinical tumor models and early‐phase clinical studies [100]. CLEC9A‐targeted vaccines have been combined with poly(I:C) or other TLR agonists to ensure effective DC activation. XCR1, another cDC1‐specific chemokine receptor, has been used to deliver antigen‐chemokine fusion proteins that use the natural XCL1–XCR1 axis. This approach facilitates selective delivery of antigens to cross‐presenting DCs and enhances vaccine efficacy with minimal off‐target effects. DEC205 (CD205) is a widely expressed endocytic receptor on cDC1s and cDC2s. Antigen delivery via anti‐DEC205 conjugates promotes internalization and MHCII presentation but often necessitates potent adjuvants to prevent tolerance induction. Studies have shown that DEC205‐targeted vaccines can prime CD4^+^ and CD8^+^ T‐cell responses when coadministered with CD40 ligands or TLR3 agonists [101].

In addition to targeting antigens, the functional activation of DCs *in vivo* has emerged as a complementary therapeutic approach. Agonistic monoclonal antibodies that target CD40, a member of the TNF receptor superfamily found on mature DCs, can mimic CD4^+^ T cell help by upregulating costimulatory molecules (CD80, CD86), promoting IL‐12 production, and licensing DCs for effective T‐cell priming [102]. When administered alongside tumor antigens or checkpoint blockade therapies, CD40 agonists have demonstrated synergistic antitumor effects in preclinical models and early clinical trials [95]. However, due to the off‐target activation of macrophages and endothelial cells, the systemic administration of CD40 agonists is associated with dose‐limiting toxicities, including cytokine release syndrome and hepatotoxicity. Current efforts emphasize engineering tumor‐targeted or conditionally active CD40 agonists or intratumoral delivery to minimize systemic exposure and enhance therapeutic indices.

Overall, *in vivo* DC‐targeting strategies demonstrate significant promise as next‐generation cancer vaccines. By leveraging the inherent antigen‐presenting capabilities of endogenous DC subsets—particularly cDC1s—these methods aim to elicit more potent and durable antitumor immunity while addressing the manufacturing challenges associated with conventional DC‐based therapies.

### Enhancing dendritic cells' function

Effective DC‐based immunotherapies depend not only on antigen presentation but also on the ability of DCs to mature, migrate to lymphoid organs, and maintain productive interactions with T cells. Several strategies have been developed to enhance these critical functions through cytokine support, combinatorial regimens, and molecular modulation of DC activity.

A promising strategy for activating cDC1 involves TriMix, a mixture of mRNAs encoding CD70, CD40L, and a constitutively active TLR4, designed to enhance the antigen‐presenting function of cDCs [103]. In clinical settings, TriMixDC‐MEL—autologous DCs electroporated with TriMix and melanoma antigens—has demonstrated safety and immunogenicity in advanced melanoma [104]. When combined with the CTLA‐4 antibody ipilimumab or administered postsurgery, this approach has resulted in durable tumor responses in patients. Other strategies to boost DC activation and recruitment *in vivo* include STING agonists (which stimulate type I interferon production and DC maturation), TLR ligands (e.g., poly(I: C), CpG, which induce pro‐inflammatory cytokines and upregulate costimulatory molecules), and FLT3 ligand (FLT3L) administration (which expands endogenous DC progenitors, particularly cDC1s, and enhances immune infiltration into tumors) [22]. Therapeutically expanding DCs, especially cDC1s, with soluble FLT3 ligand (sFLT3L) has shown potential in boosting antitumor immunity. In preclinical models, sFLT3L alone or in combination with radiotherapy, CD40 agonists, or TLR3 agonists (e.g., poly(I:C)) improved tumor control, enhanced CD8^+^ T‐cell cross‐priming, and in some cases triggered abscopal effects. Clinical trials have also evaluated sFLT3L in conjunction with poly(I:C) and antigen‐targeting antibodies (e.g., DEC205–NY‐ESO‐1), leading to increased DC expansion and antigen‐specific T and NK cell activation in patients with melanoma (NCT02129075). Despite these advantages, FLT3L also expands Tregs, which may limit its effectiveness. Moreover, in a phase I trial (NCT01811992), GS‐3583 (FLT3L‐Fc) was well tolerated but resulted in fatal acute myeloid leukemia (AML) in a patient with preexisting clonal hematopoiesis, underscoring the need for caution in patient selection.

Exogenous cytokines such as GM‐CSF, IL‐2, and type I interferons (IFN‐α/β) have been widely used to enhance DCs' survival, maturation, and T‐cell priming capabilities. In particular, GM‐CSF is used *in vitro* to generate monocyte‐derived DCs and *in vivo* to support myeloid lineage expansion. IL‐2 has been used to promote the proliferation of antigen‐specific T cells following DC activation, while IFN‐α enhances antigen presentation and IL‐12 production by DCs. However, the systemic administration of these cytokines often leads to off‐target toxicity and unwanted immunopathology. To address this issue, tumor‐localized cytokine delivery systems are being developed, including nanoparticle‐based carriers that co‐deliver cytokines with tumor antigens, oncolytic viruses engineered to express cytokines *in situ*, and hydrogel depots that release cytokines in a sustained, tumor‐restricted manner. These platforms aim to improve local immune activation while minimizing systemic side effects, which is a significant limitation of earlier cytokine‐based therapies.

Together, these interventions aim to overcome the immunosuppressive TME, improve antigen presentation, and enhance both the quantity and quality of DC–T‐cell interactions—an essential step toward effective, long‐lasting tumor control.

### Tumor‐dendritic cells reprogramming approaches

A paradigm‐shifting strategy in cancer immunotherapy has emerged with the advent of *in situ* DC reprogramming, a technique that aims to convert nonimmunogenic tumor cells into functional, antigen‐presenting DC‐like cells. This novel approach leverages transcription factor–based cellular reprogramming, enabling tumor cells to serve as both the source of tumor antigens and the platform for immune activation. Recent work by Guo *et al*. demonstrated that the forced expression of three key transcription factors—PU.1, IRF8, and BATF3—can reprogram murine melanoma and lung carcinoma cells into DC‐like cells (termed “induced antigen‐presenting cells” or iAPCs) [105]. These reprogrammed cells acquired hallmark features of cDC1s, including upregulation of MHC class I and II molecules, costimulatory molecules (CD80, CD86), and antigen‐processing machinery. Importantly, they could cross‐present tumor‐derived antigens and prime antigen‐specific CD8^+^ T cells, leading to effective tumor clearance in immunocompetent mouse models. This strategy offers several conceptual and translational advantages, as it bypasses the need for exogenous antigen identification and delivery, integrates cell therapy and gene therapy—allowing tumor‐intrinsic and localized immune activation—and amplifies the immune response at the tumor site, potentially overcoming barriers posed by poor DC infiltration or dysfunctional endogenous DCs. In addition to engineered reprogramming using viral vectors or mRNA delivery, nonviral delivery platforms—such as lipid nanoparticles and biodegradable polymers—are under development to enhance the safety and clinical feasibility of this approach. Overall, DC reprogramming represents a disruptive innovation that transforms tumor cells into self‐targeting immune catalysts and offers a promising new direction in the innovation of cancer immunotherapy.

### T–dendritic cells bispecific antibodies

A rapidly emerging strategy in cancer immunotherapy involves using bispecific antibodies designed to engage both DCs and T cells simultaneously, thereby facilitating more efficient antigen cross‐presentation and T‐cell activation. This approach seeks to mimic and amplify the natural immunological synapse between cross‐presenting cDC1s and CD8^+^ cytotoxic T lymphocytes, which are essential for effective antitumor immunity but are often impaired in the TME. These bispecific constructs function by physically bridging DCs and T cells, overcoming spatial limitations and enhancing functional interactions. For example, one innovative design links CLEC9A, a surface receptor expressed specifically on cDC1s, with PD‐1, an inhibitory receptor on exhausted CD8^+^ T cells [106]. This CLEC9A–PD1 bispecific T‐cell engager (BiCE) promotes direct contact and reactivation of dysfunctional T cells within the TME, resulting in enhanced proliferation, effector function, and tumor clearance in preclinical models. Other constructs combine antigen delivery and costimulatory signaling in a single molecule. A notable example is a bispecific antibody targeting CD40 and DEC‐205, which engages DEC‐205 on DCs for antigen uptake and CD40 for maturation. This design delivers both signal 1 (antigen) and signal 2 (costimulation) to prime T cells more effectively [107]. Similarly, a PD‐L1 × CD3ε bispecific engager has been shown to redirect T cells to DCs and potentiate TCR signaling and cytotoxic activity [108]. The therapeutic appeal of these molecules lies in their ability to enhance cross‐priming, rescue T‐cell function in immunosuppressive environments, and enable localized immune activation, potentially reducing systemic toxicity. Thus, these bispecifics are particularly relevant in tumors with poor T cell infiltration or where conventional checkpoint inhibitors fail due to a lack of effective antigen presentation. However, translating this strategy to the clinic presents several challenges, including selectivity for cDC1s to avoid off‐target activation and to minimize toxicity. Optimizing binding affinities, half‐life, and tissue distribution will also be key to balancing efficacy and safety. Currently, most T–DC bispecific antibody platforms remain in preclinical development, but they represent a promising frontier in the rational design of immune‐engaging biologics.

## Conclusion and perspectives

Dendritic cells (DCs) are fundamental regulators of the tumor–immune interface. Their unique ability to integrate innate sensing with adaptive immune programming—through antigen presentation, costimulatory signaling, and cytokine release—places them at the center of cancer immunobiology. As detailed in this review, DCs are not a homogeneous population. Instead, they comprise a spectrum of specialized subsets—including cDC1s, cDC2s, pDCs, monocyte‐derived DCs (moDCs), transitional DCs (tDCs), and DC3s—each with distinct ontogenies and functional identities shaped by local cues and pathological context [2, 19].

Within tumors, the functional behavior of DCs is particularly dynamic. While subsets like cDC1s are crucial for cross‐priming CD8^+^ T cells and promoting cytotoxic immunity, many DCs are functionally suppressed by tumor‐derived factors such as IL‐10, TGF‐β, VEGF, and metabolic stressors such as hypoxia and lactic acid. These signals can disrupt DC maturation, inhibit IL‐12 production, and reduce CCR7 expression, impairing their migration to lymph nodes and attenuating their ability to activate T cells [24, 109]. In some cases, DCs adopt tolerogenic states characterized by the expression of checkpoint ligands, anti‐inflammatory cytokines, and poor immunostimulatory capacity, contributing to immune evasion rather than activation.

This phenotypic and functional plasticity is exemplified by tumor‐associated DC subsets such as mregDCs and ISG^+^ DCs, which reflect stable reprogramming under chronic inflammatory or suppressive conditions [110, 111]. In parallel, newly described populations such as DC3s and tDCs blur the boundaries of classical DC lineages and suggest that inflammation, cytokine signaling, and epigenetic remodeling can reshape DC fate and function [17, 20].

The recent identification of RORγt^+^ APCs in mucosal tissues, which induce regulatory T cells via PRDM1, IL‐10R, and CCR7 expression, further expands the conceptual landscape. While these cells have not yet been observed in tumors, their molecular profile resembles that of tolerogenic DCs found in epithelial cancers, raising the possibility that tumors might co‐opt developmental programs evolved for peripheral tolerance [112, 113].

Therapeutically, DCs are being harnessed through a variety of strategies, including *ex vivo*–loaded vaccines, *in vivo* targeting of DC‐specific receptors (e.g., CLEC9A, DEC205), and mRNA‐based delivery platforms. Cutting‐edge approaches such as induced APCs (iAPCs)—which reprogram tumor cells to adopt DC‐like properties—also hold great promise [114]. Nevertheless, clinical outcomes remain inconsistent, underscoring the need for deeper insights into the biology of DC dysfunction within tumors.

Translating this knowledge into effective and durable cancer therapies remains a major challenge. One critical question is whether the dysfunctional or immunoregulatory states observed in tumor‐infiltrating DCs—such as mregDCs or ISG^+^ DCs—can be reversed *in situ* without disrupting systemic immune balance. The stability and plasticity of these states, particularly under the metabolic and cytokine pressures of the TME, are not yet fully understood. Emerging DC subsets such as RORγt^+^ APCs, tDCs, and DC3s further complicate this landscape, raising the need for improved functional markers and context‐specific targeting strategies.

Moreover, patient heterogeneity and tumor immune architecture present major obstacles to broad clinical translation. Key outstanding questions include: How can we define biomarkers predicting the response to DC‐targeted therapies? What are the dominant suppressive circuits preventing DC maturation or antigen presentation in specific tumor settings? And importantly, can we rationally design interventions that restore DC function without eliciting off‐target inflammation or tolerance breakdown?

Future progress will depend on integrating spatially resolved and temporally dynamic data from preclinical models and patient samples. High‐dimensional single‐cell profiling, in combination with functional genomics and *in vivo* perturbation studies, will be essential to map actionable DC states and their interactions with T cells, stromal elements, and metabolic cues. Ultimately, realizing the therapeutic promise of DCs will require not only overcoming their suppression in cancer, but leveraging their plasticity and versatility to design more precise and durable immunotherapies.

## Author contributions

ECSB, DR, GM, and MG contributed equally to the conceptualization, literature analysis, and drafting of the manuscript. MG supervised the project and critically revised the final version of the manuscript for intellectual content. All authors read and approved the submitted version.
